# Perceptions, people and place: Findings from a rapid review of qualitative research on youth gambling

**DOI:** 10.1016/j.addbeh.2018.10.008

**Published:** 2018-10-09

**Authors:** Heather Wardle

**Affiliations:** London School of Hygiene and Tropical Medicine, 15-17 Tavistock Place, London WC1H9SH, United Kingdom

**Keywords:** Gambling, Youth, Review, Qualitative

## Abstract

Exploring perceptions, experiences and determinants of youth gambling is crucial for understanding both the impact of youth gambling now and the antecedents of future behaviour. Qualitative research plays an important role in exploring these processes, yet to date, there has been no systematic review of qualitative scientific literature of youth gambling behaviour. A rapid review of three databases (PubMed, Scopus, Web of Science) and grey literature was conducted to map what qualitative research has been conducted, to identify gaps and discern emerging theories or themes about youth gambling behaviour. Peer-reviewed studies were included if they qualitatively explored youth experiences or perceptions of gambling. Narrative and thematic synthesis identified key descriptive and analytical themes covered by the studies. From 75 studies, 21 papers were included. Studies focused on youth perceptions of gambling (including advertising) and/or the factors shaping behaviour. Those which examined perceptions highlighted the normalcy of gambling in the minds of youth and its embeddedness in everyday life but also ambiguity and nuance around their differing perceptions of what gambling is. Studies emphasised the relationship between people (family, peers), place, technology and advertising as key facilitators of behaviour. This review shows young people’s perceptions of gambling differs from legal and legislative definitions, which risks underestimating the nature and extent of youth gambling behaviour. There are also notable gaps in knowledge, specifically around the role of technology in shaping gambling behaviours beyond consideration of access and availability. There is a pressing need to better understand the whole techno-ecosystem in which gambling is situated and young people’s relationship with it to understand youth gambling.

## 1 Introduction

Gambling is increasingly recognised as a public health issue, contributing to ongoing health inequalities, the experience of physical and mental health problems and poor wellbeing (Lancet, 2017). The impacts of gambling can endure, affecting the health and wellbeing of individuals, families and communities (Langham et al., 2016). Gambling among children and young people is common, despite gambling in most jurisdictions being a legally age-restricted activity. In Great Britain, 12% of 11–15 year olds gambled in the past week, over half of which did so on age-restricted and commercial forms of activity (Wardle, 2018). Studies of youth gambling have shown inequalities in youth gambling behaviour, with those from more socially deprived backgrounds being more likely to experience problems (Blinn-Pike, Worthy, & Jonkman, 2010). Studies also suggest that gambling problems among children and young people are at least as common, if not more so, as among adults. In Britain, 0.9% (around 31,000) children aged 11–15 are estimated to be problem gamblers (Wardle, 2018).

Gambling in youth is a key predictor of future problems among adults (Blinn-Pike et al., 2010). These patterns, combined with rapidly changing technological, regulatory and corporate practices, has led to increasing concern about the impact of gambling upon the lives of young people. This is both in terms of the immediate impact of youth gambling but also as the antecedents of future behaviour (Responsible Gambling Strategy Board, 2018). It is therefore important that the gambling experiences of children and young people are better understood to develop insight into the ways behaviours are initiated, embedded and, crucially, change over time. To better understand the place and impact of gambling in the lives of young people, qualitative research methods are needed to explore the meaning and practices of gambling among youth and the contexts which shape these experiences. To date, much of our knowledge about children’s and young people’s gambling behaviour has been generated from quantitative research, exploring how many children gamble, their motivations and attitudes toward gambling and their experience of problems (Valentine, 2016). These studies have highlighted early onset of gambling, poor levels of academic attainment and disrupted family relationships as key risk factors for gambling problems. They have also noted associations between gambling and low self-esteem, high levels of extroversion and poor self-discipline, drug and substance misuse, experience of depression and anxiety and suicide and suicidality among youth (Blinn-Pike et al., 2010).

In a search conducted by the author, no systematic reviews of qualitative research on youth gambling were identified to date neither have prospective ones been registered on sites such as Prospero. In 2010, Blinn-Pike et al. conducted a literature review on adolescent gambling and identified only eight qualitative studies, which were excluded from their review because of space limitations (Blinn-Pike et al., 2010). Other reviews have noted the existence of qualitative research but not attempted to synthesise findings between them (Valentine, 2016). In recent years, there has been marked increase in the amount of qualitative research conducted on the gambling behaviours of young people and children. Indeed, there has been an increase in use of qualitative techniques to explore gambling generally. Reith and Dobbie’s seminal work with gamblers in Glasgow, a qualitative assessment of gambling behaviour over a five year period, highlighted the dynamic nature of gambling behaviours, emphasising the importance of social networks, context and life events in the initiation, maintenance and change of behaviours (Reith & Dobbie, 2013). Breen and Hing’s investigation of gambling behaviour across the life course of indigenous Australians emphasised the dynamic interplay between social contexts and individual choices in shaping gambling behaviours and trajectories (Breen & Hing, 2014). These works provide a much needed corollary to quantitative studies of gambling behaviour, helping to unpack the complex interplay of factors that underpin behaviour changes and exploring the lived experiences of individuals.

Qualitative synthesis is a powerful tool bringing together findings and insights from a range of disparate studies. Synthesis of qualitative research across independent studies can help to generate more comprehensive and generalizable theories, identify new themes and concepts, deepen understanding drawn from individual studies and thus strengthen their assertions. With regards to youth gambling, synthesis may allow us to learn more about the ways that young people view gambling, the implications this has for policy practice and its relationship with future behaviour. This may allow us to form new theories about how gambling fits into the lives of young people, how they navigate and understand complexity and their reactions and response to the rapidly changing techno-ecosystem of which gambling is a part. Such reviews also have practical importance, providing an overview of evidence on which interventions and policy decisions might be made.

The aim of this paper is to provide the first synthesis of key themes and concepts identified from qualitative investigations of youth gambling behaviour in order to map what research has been done and identify gaps. This paper provides an initial narrative overview of the key themes emerging and starts to identify potential new theories and concepts for future research.

As the primary objective was to map the qualitative research landscape in order to identify gaps and emerging theories and themes, a rapid review of research evidence was conducted. Rapid reviews draw on systematic review methodologies, with modifications, to conduct reviews within a shorter time frame and typically include fewer studies than full systematic reviews (Haby et al., 2016).

## 2 Methods

### 2.1 Rapid review methods

A systematic rapid review was conducted to identify relevant studies. In systematic rapid reviews, the procedures used are set out apriori in a rapid review protocol, in this case following the procedures set out by Haby et al., (2016) (see Appendix 1). The protocol for this review documents the search terms to be used for all databases (combinations of different descriptors for children and young people, gambling and qualitative research) and the databases searched (PubMed, Web of Science, Scopus) as well as grey literature (searched via GambleAware’s Infohub, the British Gambling Commission’s website and GambLib (the Gambling Research Library)). The protocol also outlines the inclusion criteria (any peer reviewed study using qualitative methods to explore gambling perceptions and behaviours among youth up to August 2017, when the review was conducted); the exclusion criteria (English language only, focus on OECD countries, not evaluations of interventions or experiments); the types of participants (up to the age of 24, where studies spanned this cut-off they were included); data extraction methods; quality control (all studies were reviewed against the Centre for Appraisal Programme checklist for qualitative research, used to exclude studies of lower methodological quality. As there is no definitive criteria of what counts as quality, studies were included if they at least contain credible and clear findings); and synthesis methods.

### 2.2 Synthesis

Narrative synthesis of key themes across studies was used to identify key themes and concepts from the review articles. All eligible studies were read and reread and key themes listed for each. In total, 17 different themes were noted from the studies. Once identified, themes and concepts were compared between studies. Each study was reread and notes, verbatim quotes and findings relating to each theme entered into a thematic framework. This technique has been used previously to help synthesise findings when a large number of studies with disparate aims are involved (Atkins et al., 2008). Because the themes included in the framework emerged from review of the primary data, rather than prior knowledge, this remains an inductive approach (Atkins et al., 2008).

## 3 Findings

### 3.1 Search results

**Fig. 1:**
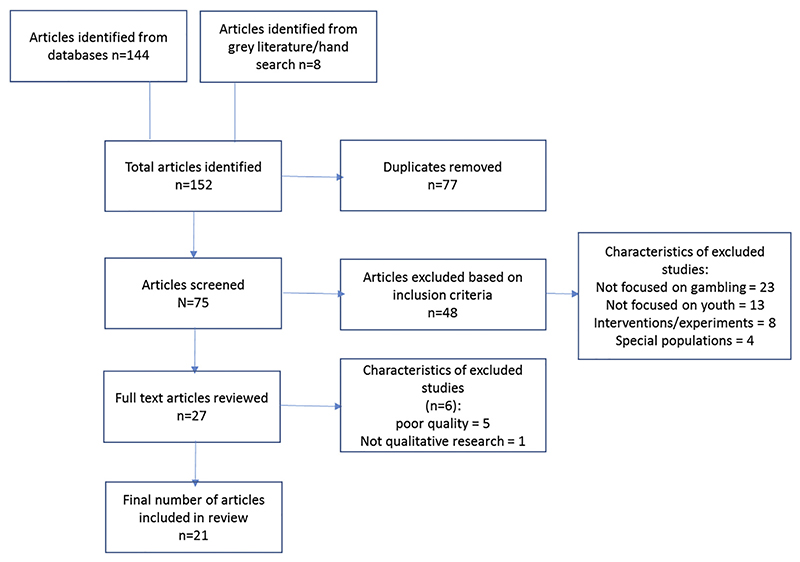
study selection flow chart.

Once duplicates were removed, 75 studies were identified and screened against the inclusion criteria. Of these 27 studies met the criteria, though a further six were subsequently identified as ineligible based on full review of the text. Data were synthesised from 21 papers, representing 16 unique studies (see Fig. 1 for full search details).

### 3.2 Characteristics of the studies

Table 1 summarises the characteristics of the papers included in the review. Of these, four were published prior to 2010, the rest being published since then, with a notable increase in number from 2015 onwards. Eight were based on fieldwork conducted in Australia, five from Canada, three from Denmark and England respectively and one each from Belgium and Portugal. There was a broadly even split between studies using in-depth interviews (either face to face or over the phone) or focus groups as their primary methodology; only one used observations. Seven studies focused on young adults aged 18 and over, with a maximum age of 37 in some cases. Others focused on adolescents (broadly those aged between 14 and 20) or children aged under 16 alone.

### 3.3 Key themes

The themes included in these studies covered two interrelated topics: exploration of young people’s perceptions and meaning of gambling and factors that influence behaviour.

#### 3.3.1 Theme 1: perceptions and meaning of gambling

##### 3.3.1.1 Normality

Across most studies, gambling was viewed as part of the fabric of everyday life, that *“it’s so much a part of normal life, it’s almost what everyone does”* (Deans, Thomas, Daube, & Derevensky, 2016b) or that *“it’s the kind of the thing you have to do at least once”* (Pitt, Thomas, Bestman, Daube, & Derevensky, 2017). In some cases, gambling was a rite of passage:

*“it was my 16^th^birthday so I could, so I thought that I just did. It was the first time I did it and I have never done it again”* (Carran & Griffiths, 2015).

*“The time just after you turn 18, you want to go there [the casino]”*(Kristiansen & Trajberg, 2017).

For others, gambling was so deeply embedded within the rituals of everyday life that some youth queried whether it was gambling at all:

*“when I get the paper I always pick up a Tattslotto ticket, do you class that?”* (Nekich & Ohtsuka, 2016)

For many, gambling was embedded within the practices and norms of social life, being something that was shared within families or peer groups (Pitt et al., 2017). Other studies noted how technology, particularly mobile apps, underpinned this sense of normalisation with gambling now being a part of social practice and ritual for some groups:

*“We’ll pull out our phones, we’ve all got a separate app, and we’ll bet on like first try scorer”* (Deans et al., 2016b).

Within the Australian studies, there was emphasis on the reciprocal relationship between sports and gambling and its role in positioning gambling as a normal activity, where young men’s enjoyment of sport was reframed through the lens of gambling (Deans et al., 2016b; Deans, Thomas, Derevensky, & Daube, 2017). This was replicated in a Danish study, where Kristensen et al. observed that it was no longer enough to simply support your team, but that you had to demonstrate allegiance through betting (Kristiansen, Reith, & Trajberg, 2016).

Some studies explored the role of advertising and marketing in normalisation, with some finding that adverts made gambling seem *‘harmless’*, *‘fun’* (Korn, 2005) or that *“the more you see it [the adverts], the more you think that’s okay”* (Deans et al., 2017).

One study found that whilst gambling may be commonplace or ‘normal’ in society generally, this was not the case for all cultural groups; Canadian Polish youth stated that gambling was frowned upon within their community and not part of family or social life (Zangeneh, Mann, McCready, & Osen, 2010).

##### 3.3.1.2 Nuance and ambiguity around perceptions of gambling

Across most studies, young people could typically identify and articulate popular types of gambling, though many studies did not appear to explore this from the perspective of youth, assuming that knowledge or views about what constitutes gambling were similar to those held by the authors. Studies which explored this specifically found more nuanced and ambiguous views of what gambling was. In some cases, gambling was seen as an extension of dares or bravado (Skinner, Biscope, Murray, & Korn, 2004), or informal betting:

*I bet my dad’s friend 10 push ups if Geelong would beat the Western Bulldogs. I won. I also bet my Nana 10 push ups that Tom Hawkins or Daniel Menzel would score first* (Pitt et al., 2017).

For some, there was a sense that gambling was risking something you value, not necessarily money, such as *“putting something you value on the line to see if you can gain more”* or *“betting your money or anything”* (Korn, 2005), with a distinction between formal or ‘serious’ gambling, where you bet money, and informal or ‘friendly’ gambling, where you bet things you value in the hope of reward (Korn, 2005; Pitt et al., 2017). ‘Serious’ betting tended to involve money:

*“you have to play for money, because if you don’t, then people do not take the game seriously”* (Zaman, Geurden, De Cock, De Schutter, & Abeele, 2014)

Gambling was seen by some as synonymous with risk and it was debated whether things like buying insurance constituted gambling or not (Korn, 2005). In other studies, youth perceptions of gambling were clearly linked to financial risk, whereby gambling involved the risk and reward of money (Caldo, Alexandre, & Griffiths, 2014; Carran & Griffiths, 2015).

*“on gambling the motivation is only winning money, that’s it”* (Caldo et al., 2014)

Ambiguity about what constituted gambling was notable when young people discussed their perception of online social media gambling and play for free games. There was little consensus evident within or between studies as to whether children and young people considered gambling and betting for virtual money or prizes to be ‘real’ gambling.

When asked to describe different types of gambling, some youth included free online games *“well, there’s some poker that you play for free and there’s the member area where you play for money”* (Wilson & Ross, 2011) and others saw the functionality for ‘betting’ within video games as the same as gambling (Caldo et al., 2014). Some participants were clear that gambling for virtual chips, points or virtual prizes was different from gambling for real money (Carran & Griffiths, 2015) whereas others felt they were similar in that things of value were being risked and rewarded:

*“gaming and gambling are very similar&there are MMORPGS which at the end of a given tournament we can win money”* (Caldo et al., 2014).

There was only one study which was unambiguous on this, stating that youth clearly saw these as different types of activity (Carran & Griffiths, 2015).

This general sense of ambiguity was evident in some accounts of online gambling. Three of the eight studies which looked at online gambling noted how the online environment and platform made ‘for money’ gambling seem less real, less tangible (Deans, Thomas, Daube, & Derevensky, 2016a; Kristiansen & Trajberg, 2017; Zaman et al., 2014). In some cases, this disassociation was because of the lack of physical hand over of money, with the monetary numbers displayed within accounts being viewed as symbolic figures, *‘just numbers on a screen’*, not real money (Deans et al., 2016a). For others, it related to the speed of play and because online gambling felt less real, they ended up *‘playing them [online slots] really fast’* (Kristiansen & Trajberg, 2017).

##### 3.3.1.3 Motivations

Most studies explored motivations for gambling. These varied between asking youth about their perceptions of why other people gambled to asking about their own gambling motivations. Those that focused on the motivations of youth themselves tended to coalesce around the social importance of gambling (Korn, 2005; Kristiansen et al., 2016; Kristiansen, Trajberg, & Reith, 2015; Nekich & Ohtsuka, 2016; Skinner et al., 2004), though the hope of winning money also featured as a common motivation (Caldo et al., 2014; Carran & Griffiths, 2015; Wilson & Ross, 2011; Woods & Griffiths, 2002; Zaman et al., 2014). In some studies, gambling was seen as a way to assert social standing and an *‘opportunity to gain prestige by winning’* (Kristiansen et al., 2016).

Fear of losing was a prominent theme across studies as to why some young people did not gamble: *“kids our age don’t have access to money”* (Skinner et al., 2004) or *“I don’t want to be one of those poor people”* (Bestman, Thomas, Randle, & Pitt, 2017). The idea of losing what little money they had was particularly challenging, but in some cases this was underpinned by a broader fear of what this might lead to:

*“I know with pokie machines, you put money into them and then if you lose you have to keep putting money in them - until you’re poor* (Pitt et al., 2017)

For others, it was underpinned by wanting to use their money for other things like *‘a dog, house or car’* (Pitt et al., 2017) or *‘also that I actually like to have money for other things besides gambling, and then there is the colossal disappointment when I lost all that money, so I just don’t feel like gambling anymore”* (Kristiansen et al., 2016).

One study explored gender differences around the fear of losing, with girls arguing that they gamble less because they are smarter with money and are less interested in *‘giving their money away’*. Whereas boys felt they had greater disposable income, so fear of losing was less of a concern to them (Wilson & Ross, 2011).

#### 3.3.2 Theme 2: factors influencing behaviour

##### 3.3.2.1 Families

Across the studies, it was clear that gambling was used as a resource within some families to create shared connections and bonds (Kristiansen et al., 2015; Kristiansen et al., 2016; Nekich & Ohtsuka, 2016; Pitt et al., 2017; Woods & Griffiths, 2002). This bonding experience engendered feelings of pride among youth, *“proud that he would let me try it, it was his money not mine that we wagered”* (Kristiansen et al., 2015). Gambling within families also transcended age boundaries and it was argued that memories of gambling within families created a sense of nostalgia, comfort and normality (Nekich & Ohtsuka, 2016). Part of this shared connection also provided a learning environment for youth, in which the rules of the games were taught by older family members to younger ones. One study highlighted how this process was gendered, with girls being introduced to games of chance by older familial females and boys being introduced to betting and games of skill by fathers and brothers (Kristiansen et al., 2016).

However, across these studies, not all had family environments in which gambling was a common activity or source of bonding. One study noted that for some children views of gambling were conditioned by conversations within their families emphasising gambling as a *“waste of money”* (Pitt et al., 2017). Others explored how in certain ethno-cultural communities gambling was not an acceptable social practice (Zangeneh et al., 2010).

##### 3.3.2.2 Peers

Friends and peer networks were powerful social groups which many studies noted shaped the gambling behaviour of young people (Deans et al., 2016b; Deans et al., 2017; Korn, 2005; Kristiansen et al., 2015; Kristiansen et al., 2016; Nekich & Ohtsuka, 2016; Woods & Griffiths, 2002). As among families, gambling was a resource used to create social bonds and connections within peer groups. In many studies, young people described how gambling within peer groups was common and that this prompted them to start gambling themselves. They gambled to ‘fit in’ or to ‘hop on the bandwagon’ (Korn, 2005; Kristiansen et al., 2015). The influence of peers in many cases seemed innocuous, with youth thinking that other people were doing it so they might as well:

*“They [my friends] were going down to place the bets on Oddset and thought that I might as well join them and try it”* (Kristiansen et al., 2015)

*“If you go into the CO-OP and all your mates are buying then, then you might feel left out, so you would buy one as well”* (Woods & Griffiths, 2002)

Gambling was seen by some as a friendly activity between peers, which served to ‘spice up’ social life and created social connectedness (Korn, 2005). However, some youth feared being marginalised, knowingly or unknowingly, by their social group if they did not gamble (Kristiansen et al., (2015), Deans et al., (2016b)). For some, the potential benefit of social belonging outweighed the financial risks as they [friends when gambling] ‘had a nice time together anyway’ (Korn, 2005). The Australian studies explored this fear of marginalisation in relations to male sporting cultures, where talk of gambling permeated social gathering around sports events (Deans et al., 2016b). To participate in the conversation, one had to talk about gambling.

As with families, peer influences could also restrain some youth from gambling, with one study reporting that changes in peer groups reduced young people’s interest in gambling, especially if the new groups were not as interested in gambling (Kristiansen et al., 2016).

##### 3.3.2.3 Places

Finally, a few studies specifically looked at how ‘place’ shaped gambling behaviours (Bestman et al., 2017; Kristiansen et al., 2015; Kristiansen et al., 2016; Kristiansen & Trajberg, 2017; Nekich & Ohtsuka, 2016; Wilson & Ross, 2011). This was generally analysed through the lens of access and availability. Easy accessibility to gambling within the local environment, such as convenience stores selling lottery tickets or electronic gaming machines being placed in bars and clubs, were described by youth as creating opportunities for gambling, regardless of whether this was intended when first visiting the venue:

*“They [my friends] don’t specifically go down to the pub with the intention of betting but if we are there, they might put money in the pokies”* (Nekich & Ohtsuka, 2016)

In some cases, place contexts interacted with peer groups and participants described groups of friends going together to local shops to gamble or that groups of boys were more likely to gamble because they were more likely to visit bars where gambling machines were located (Skinner et al., 2004).

##### 3.3.2.4 Technologies

Whilst gambling online made the activity feel less tangible, there was a notable theme relating to the incursion that smart phone gambling has made into everyday life – meaning that people can gamble ‘anywhere, anytime, anyplace’ (Pitt, Thomas, & Bestman, 2016) with some studies reporting how young people use these apps in situations and forums in which previously would not be possible:

*“Well, I always just put in 100 kroner for example, if I’m at school, and I get a little bored ha-ha, then I can just play roulette online”* (Kristiansen & Trajberg, 2017).

*“I can promise you that if I didn’t have an app on my phone, I would gamble only very rarely. If I didn’t have the app, I don’t think I would even gamble.”* (Deans et al., 2016a).

Some studies looked specifically at the relationship between social media style gambling games and ‘for money’ gambling, particularly around whether the former leads to the latter. There was a sense that those who played social media gambling style games were less serious or competitive than those who gambled for money (Carran & Griffiths, 2015; Zaman et al., 2014) and that the likelihood of transitioning would depend on individual’s motivations. That said, there was a broad perception that social media gambling games could act as a learning environment, where people learn the rules of the game (especially poker) in a less risky environment: *“Facebook is just a place to learn. To learn how to gamble”* (Kim, Wohl, Gupta, & Derevensky, 2016).

Across the studies, none of the participants said that playing these games online had encouraged them to gamble ‘for money’ though they could see the potential appeal:

*“like if you get good at it, you might feel confident, you might wanna go and play it for money”* (Skinner et al., 2004)

Others talked about the experiences of people they knew where this had happened:

*“they had a hard time with the transition from playing online for free, to paying for playing online; and they were losing like hundreds of dollars”* (Kim et al., 2016)

However, there were others, both within and between studies, who felt that these games did not replicate ‘real’ money gambling and that these games had a distinct attraction of their own (Kim et al., 2016; Kristiansen et al., 2016).

##### 3.3.2.5 Advertising and marketing

Some studies looked at young people’s responses to advertising and marketing (Deans et al., 2016a; Deans et al., 2017; Korn, 2005; Kristiansen et al., 2015; Pitt et al., 2017; Pitt, Thomas, & Bestman, 2016; Pitt, Thomas, Bestman, Stoneham, & Daube, 2016; Zaman et al., 2014), with participants noting that they were influenced, in particular, by bonus offers. As a result, some participants reported signing up with a number of operators to get these offers (Deans et al., 2017). Even among those who considered themselves to be semi-professional, these offers were seen to be *‘free money’* and incentivised participation (Zaman et al., 2014). Some described the adverts *“luring me in, in the way that if I put in 100, then I get double”* (Kristiansen & Trajberg, 2017). Others felt it would be *‘silly’* not to take up these offers (Deans et al., 2017). Related to technology, others felt they were being pursued by gambling operators who used insight data about them to personalise offers (Kristiansen & Trajberg, 2017). For some children, the advertising made them feel like they wanted to bet and if they didn’t they were missing out: *“the ads make you want to bet”* (Pitt, Thomas, Bestman, Stoneham, & Daube, 2016). This was closely linked to the perception that increasing advertising had changed the normative environment for youth, promoting a sense that it was normal, acceptable and that everyone gambles (Deans et al., 2017; Pitt, Thomas, Bestman, Stoneham, & Daube, 2016).

## 4 Discussion

To the best of the author’s knowledge, this is the first time qualitative research on young people and gambling has been reviewed and synthesised. The published research tends to focus on two related areas: young people’s perceptions of gambling and the factors that influence gambling behaviour. Key concepts cut across both, such as a role of families, peers, advertising and technology in shaping the normative environment in which young people’s perceptions are formed but also as direct influencers and facilitators of gambling behaviour.

However, there are many notable gaps in the evidence base. Exploration of social context has broadly been confined to the examination of people and place, with the latter examined through the lens of access and availability of gambling. Across studies (barring one or two exceptions) there was little exploration of gambling through the lens of gender, exploring differential perceptions and behaviours for boys and girls. There has also been little consideration of socio-economic contexts, ethno-cultural contexts or the influence of environment beyond access and availability in shaping gambling perceptions and experiences. Given that gender, class and environment have been highlighted as key factors influencing the broader experiences and transitions of young people, this is a major omission (Furlong & Cartmel, 2007).

Resources emerges as a theme that cuts across many of papers reviewed. Arguably, different perceptions of resources and the way youth think about and value things helps to explain evident ambiguity about gambling. Many of the studies did not explore perceptions of gambling from the perspectives of youth themselves but assumed a shared understanding with the study researchers. In these instances, this frames gambling through the lens of the researcher, and, relatedly, replicates dominant policy and legal paradigms within the canon of research (for example, gambling being the risk of money). By looking across the studies, it emerged that young people had a more nuanced perception of what gambling was and that, in some cases, this was underpinned by the types of resources available to them. In the context of young people’s lives, especially younger children, who have more limited access to monetary resources, gambling could involve the risk and reward of items which have personal value to them, which may have greater personal value than their monetary worth. Few studies explored this (though there were some exceptions). This has implications for how we view gambling behaviour among young people. In Great Britain, the annual study of youth gambling asks children to report what activities they have spent money on (Gambling Commission, 2016). Whilst this may replicate legal definitions of gambling used in British legislation, this focus may be missing a greater range of practices that young people engage in and thus underestimate the breadth of youth gambling.

Indeed, this is ambiguity is increasingly recognised by legislators and regulators themselves. In Great Britain, money as the object of risk and reward was enshrined in the Gambling Act 2005: this defined gambling as participating in gaming, betting or lotteries. ‘Gaming’ was described as having to be gaming for a prize, whereby a prize means money or ‘money’s worth’. ‘Betting’ was subject to no such qualification though lottery prizes were less tightly defined (consisting of money, articles and services). The concept of ‘money’s worth’ introduces definitional ambiguity, as ‘money’s worth’ is inherently subjective, though a more objective meaning was no doubt intended (i.e., that prizes are worth money and that the value of that worth is known). Even so, how prizes are valued may mean different things to different people and whether the prize is indeed viewed as ‘money’s worth’ will vary. This potential difference in meaning creates legal ambiguity about what does and does not count as gambling, something which when reviewing online social casino gambling with virtual currency the British Gambling Commission has itself recognised (Wardle, 2015). This ambiguity also appears to be recognised by the young people interviewed in the studies reviewed.

Gambling itself was also used as a type of resource, especially among families and peers. Among families, it was a resource to facilitate social connectedness and strengthen bonds. Similar themes were evident among peers but gambling was also viewed as resource that, as well as fostering social connectedness, could bring prestige, foster pride and enhance social standing. Gambling among youth appears to have a wider value proposition than the risk and reward of money. Across the studies, gambling was not generally examined in the context of everyday life. Understanding how gambling is embedded within and supports broader social practices and how behaviour is influenced by broader circumstances, especially in relationship to available resources and the personal value propositions inherent within the activity, is worthy of further exploration.

These differing perspectives may go some way to explain the contested relationship between social media gambling games and real money gambling. Depending on how youth view and perceive ‘virtual’ items will likely affect their views of whether this constitutes gambling or not. This too may be further blurred by online gambling promoting a sense of action being ‘unreal’, or perhaps, as argued by Fisher (1993)), be related to gambling being viewed as a form of play. Finally, there was very little consideration of the broader techno-ecosystem that accompanies online gambling and gambling-style games. Themes of peer influence tended to focus on physical gambling within peer groups (going somewhere or doing something together, even if that was sitting in the same place with your peers but gambling online). It was less clear how these peer connections are translated and reproduced in online environments and, importantly, the implications of this for behaviour change. Transitions in peer networks can underpin transitions in gambling behaviour (Reith & Dobbie, 2013). However, it is unclear if online peer networks among youth are as fluid as physical peer groups and thus whether this creates the same opportunities for behaviour change.

## 5 Limitations

This paper inherits the limitations of the studies it reviews, the quality of which was variable. Many of the studies did not have explicit statements of how the analysis had been conducted, and some had minimal presentation of verbatim results. Others simply presented long lists of things that people had said, showing a lack of detailed analysis. This has implications for this review, as the studies are reflections of what the authors chose to present. Across all studies, the aims and objectives were disparate and some had very wide ranging aims and objectives, trading depth for breadth. This paper reviews the key themes emerging, but there are few insights into the mechanisms and contexts which underpin these observations. Few studies discussed sampling in full, and with some notable exceptions, theoretical saturation tended not to be a sampling criteria. This review may reflect some unstated and inherent systematic biases in who took part and not reflect the full diversity of opinion, behaviours and experiences (the lack of cultural and gender diversity in many of the studies reviewed makes this likely). Finally, this synthesis has further limitations relating to the rapid review methods which looks at a more limited range of databases than a full systematic review (though Haby et al. (2016)) have suggested that rapid review methods do not materially alter conclusions drawn when compared with full systematic review methodologies); articles included were in English only and because of resource constraints, only one reviewer (the author) was used for study selection, quality appraisal, data extractions and synthesis. All of this may mean that some studies have been missed from the review. Finally, the majority of evidence was generated from Australia and Canada, both of which have unique gambling environments and specific policy concerns (for example, the concern about the rise of sports betting advertising in Australia) which shapes the type and nature of evidence produced. That said, most themes noted were evident across studies from different jurisdictions. This is one of the benefits of qualitative synthesis as it allows common themes and theories which transcend national contexts to be identified.

## 6 Conclusions

This review shows that young people’s perceptions of gambling does not necessarily reflect the assumed common understanding set out by policy makers or in legal definitions. This divergence risks those involved in developing policies and practice around youth gambling underestimating its extent and influence. Gambling is viewed by youth as normal, a viewpoint promoted by advertising and marketing. The role of technology in shaping behaviours needs to be extended from focus on access and availability. For youth, ‘real’ life is increasingly ‘digital’ life encompassing a broad techno-ecosystem. We should seek to understand how this is embedded within the everyday life of young people and their networks and explore its implications for how young people view, relate to and understand gambling.

## Supplementary Material

Appendix A

## Figures and Tables

**Table 1:** Summary of papers included in the review

Authors	Year study published	Country	Age of participants	Objectives	Methods
Fisher, (1993)	1993	England	11-20	To provide a sociological account of fruit machine play among young people in a seaside arcade	Observations, groups and interviews
Woods & Griffiths, (2002)	2002	England	11-15	To explore why adolescents start gambling on lotteries and scratchcards, and to identify factors that maintain their gambling behaviour.	Focus groups
Skinner et al., (2004)	2004	Canada	10-20	To better understand how gambling is viewed by youth, their personal and social concerns about gambling and the role of technology, especially the internet.	Focus groups
Korn, (2005)	2005	Canada	13-17	To examine the possible impact of commercial gambling advertising on the knowledge, attitudes, beliefs, and behavioural intentions of youth.	Focus groups
Zangeneh et al., (2010)	2009	Canada	18-25	To explore, through the application of Problem Behaviour Theory, factors associated with gambling and problem gambling among adolescents in various ethno-cultural groups	Focus groups
Wilson & Ross, (2011))	2011	Canada	13-18	How does encroachment of Video Lottery Terminals into spaces that young people use on a daily basis influence their use of that space and their views of gambling.	Focus groups
Zaman et al., (2014)	2014	Belgium	18-24	To explore the dominant motivations endorsed for playing online poker in amateur, semi-professional and professional players and how these motivations relate to computer interface preferences.	In-depth interviews
Caldo et al., (2014)	2014	Portugal	13-26	To explore the role of gambling and gaming in young adults’ lives; general perceptions of gambling and gaming; perceived common features between both gambling and gaming; motivations underlying gambling and gaming behaviours; meta-perceptions of family members’ opinions on gambling and gaming behaviours; feelings that gambling and gaming triggered, and the potential negative outcomes of gambling and gaming.	Focus groups
Kristiansen et al., (2015)	2015	Denmark	12-20	What are the reasons for young people’s involvement in gambling activities and what role does the social context (community, peers, and social relationships) and gender play in initiatingxgambling among young people?	In-depth interviews
Carran & Carran & Griffiths, (2015)	2015	England	14-19	To examine the relationship between gambling and gambling-like games via the use of qualitative focus groups.	Focus groups
Deans et al., (2016a))	2016	Australia	Young men 20-37	Are there distinct factors that encourage young men to consume gambling products in different gambling environments? Do these factors contribute to risky gambling behaviour? Does gambling co-exist with other forms of risky behaviours in some environments?	In-depth interviews
Kim et al., (2016)	2016	Canada	Students 18-24	To explore the extent to which social media users are aware of social casino and their perceptions of them. To explore the link between social casino and online gambling.	Focus groups
Nekich & Ohtsuka, (2016)	2016	Australia	20-25	To analyse culturally shared repertories used by young gamblers to describe their gambling behaviour.	In-depth interviews
Pitt, Thomas, & Bestman, (2016))	2016	Australia	14-18	How do young people and their parents describe the relationship between gambling and sport? What factors may influence these perceptions? How do young people interpret the messages they see about gambling during sport? Is there evidence to suggest that young people are increasingly viewing sport through a ‘gambling lens’?	In-depth interviews
Kristiansen et al., (2016)	2016	Denmark	12-20	To investigate patterns of change in gambling behaviour and the complexities and social contexts, as well as the subjective meanings that lie behind such changes.	In-depth interviews
Deans et al., (2016b)	2016	Australia	Young men aged 20-37	What role does sports wagering play within peer groups and to what extent do individuals gamble on sports within peer groups? What role do social norms play in the establishment of sports wagering rituals in and among the group? Does language play a role in shaping young men’s perceptions about (and ascribed meaning toward) sports wagering?	In-depth interviews
Pitt, Thomas, Bestman, Stoneham, & Daube, (2016)	2016	Australia	8-16	To explore how children and adults recall the content and promotional channels for sports wagering marketing.	Interviews including picture board prompts and open-ended questions
Bestman et al., (2017)	2017	Australia	6-16	To what extent can children recall and describe EGMs and behaviours associated with EGM use in community venues? What factors influence and reinforce children’s perceptions of EGMs in community venues? Do children express current or future consumption intentions to use EGMs?	In-depth interviews
Pitt et al., (2017))	2017	Australia	8-16	Whether there are specific socialisation factors that positively influence children’s understanding and popularity of specific gambling products; Do some factors appear to be more influential than others in shaping children’s gambling attitudes and consumption intentions. How can public health strategies be used to reduce the harms associated with socialising agents which are particularly influential in positively shaping children’s gambling attitudes and consumption intentions?	In-depth interviews
Kristiansen & Trajberg, (2017)	2017	Denmark	12-20	To explore how young people experience and respond to changes in gambling opportunities.	In-depth interviews
Deans et al., 2017	2017	Australia	Young men 20-37	How do marketing mechanisms seek to create a cultural alignment between betting and sports? Is there evidence that marketing strategies may be influencing new betting “identities” associated with sports? 3. Do specific forms of promotions encourage young men to gamble more frequently and on events that they would not otherwise bet on? Are there specific strategies that may have the potential to reduce or prevent the risks or harms posed by the marketing for these products?	In-depth interviews

## Highlights

- There is ambiguity in how young people perceive gambling and what they do and do not count as gambling activity.
- Young people may perceive gambling differently to that of policymakers meaning youth gambling may be underestimated.
- Advertising and marketing influence the normative environment for gambling and encourage some youth to want to gamble.
- The role of technology in shaping perceptions of gambling and facilitating behaviour change has been under-explored.
