# Supplementary material for: Perceptions, people and place: Findings from a rapid review of qualitative research on youth gambling
Source: Addict Behav. Author manuscript; Available in PMC 2023 Jul 10. (PMC7614737; doi:10.1016/j.addbeh.2018.10.008)
Supplement: Appendix A [file EMS177454-supplement-Appendix_A.docx]

**Appendix A: Detailed rapid review protocol. Adapted from Haby et al [9]**

| **Research question** | What qualitative research into youth gambling behaviour has been undertaken? |
| --- | --- |
| **Methods** | |
| **Search databases:** | |
| Academic | Scopus; PubMed; Web of Science |
| Grey literature | Research reports accessed through Gamble Aware InfoHub; Gambling Commission; GambLib (Gambling Research Library) |
| **Search terms (in title/abstract)** | (Young OR youth OR children OR pupils OR students OR adolescents) AND gambling AND qualitative; (Young OR youth OR children OR pupils OR students OR adolescents) AND gambling AND focus groups; (Young OR youth OR children OR pupils OR students OR adolescents) AND gambling AND depth interviews; (Young OR youth OR children OR pupils OR students OR adolescents) AND gambling AND ethnograph*; (Young OR youth OR children OR pupils OR students OR adolescents) AND gambling AND semi structured; |
| **Inclusion criteria:** | |
| **Types of studies** | Systematic reviews and primary studies included.  Types of primary studies included: qualitative, including longitudinal qualitative. Mix method.  Types of primary studies excluded: quantitative – all |
| **Types of participant** | Young people aged 10-24 living in the general population; studies of students included. |
| **Types of articles** | Any study that uses qualitative methods to explore youth gambling behaviour.  Types of primary studies excluded: evaluations of interventions; experiments; PhD/Masters theses |
| **Types of comparisons** | N/A |
| **Types of outcome measure** | N/A |
| **Other** | English only; focus on OECD countries |
| **Data collection and analysis:** | |
| **Selection of studies** | Searches will be conducted and screened according to the selection criteria by the review author. The full text of any potentially relevant papers will be retrieved for closer examination. All studies which initially appear to meet the inclusion criteria but on inspection of the full text paper do not meet the inclusion criteria will be detailed in a table ‘Characteristics of excluded studies’ together with reasons for their exclusion. The results of the study selection process will be presented in a PRISMA format flow chart. |
| **Data extraction** | Data extracted for each article will include:   - Author - Year of publication - Year of study - Country or region of study - Objectives - Methods/Study design - Population/sample size - Results - Quality assessment* - Limitations   *see below |
| **Assessment of methodological quality** | Any systematic reviews will be reviewed against the AMSTAR criteria. Qualitative studies will be assessed using the CASP checklist. As there is no definitive criteria of what counts as quality, studies will be included if they at least contain credible and clear findings. |
| **Data synthesis** | Narrative synthesis summarising findings, key themes and concepts. Descriptive summaries about the breadth of topics addressed, methods used and key themes will be presented. Gaps highlighted. |
